# Synchrotron Reveals Early Triassic Odd Couple: Injured Amphibian and Aestivating Therapsid Share Burrow

**DOI:** 10.1371/journal.pone.0064978

**Published:** 2013-06-21

**Authors:** Vincent Fernandez, Fernando Abdala, Kristian J. Carlson, Della Collins Cook, Bruce S. Rubidge, Adam Yates, Paul Tafforeau

**Affiliations:** 1 Evolutionary Studies Institute, University of the Witwatersrand, Johannesburg, Gauteng, South Africa; 2 Department of Anthropology, Indiana University, Bloomington, Indiana, United States of America; 3 Museum of Central Australia, Araluen Cultural Precinct, Alice Springs, Northern Territory, Australia; 4 European Synchrotron Radiation Facility, Grenoble, France; Ludwig-Maximilians-Universität München, Germany

## Abstract

Fossorialism is a beneficial adaptation for brooding, predator avoidance and protection from extreme climate. The abundance of fossilised burrow casts from the Early Triassic of southern Africa is viewed as a behavioural response by many tetrapods to the harsh conditions following the Permo-Triassic mass-extinction event. However, scarcity of vertebrate remains associated with these burrows leaves many ecological questions unanswered. Synchrotron scanning of a lithified burrow cast from the Early Triassic of the Karoo unveiled a unique mixed-species association: an injured temnospondyl amphibian (*Broomistega*) that sheltered in a burrow occupied by an aestivating therapsid (*Thrinaxodon*). The discovery of this rare rhinesuchid represents the first occurrence in the fossil record of a temnospondyl in a burrow. The amphibian skeleton shows signs of a crushing trauma with partially healed fractures on several consecutive ribs. The presence of a relatively large intruder in what is interpreted to be a *Thrinaxodon* burrow implies that the therapsid tolerated the amphibian’s presence. Among possible explanations for such unlikely cohabitation, *Thrinaxodon* aestivation is most plausible, an interpretation supported by the numerous *Thrinaxodon* specimens fossilised in curled-up postures. Recent advances in synchrotron imaging have enabled visualization of the contents of burrow casts, thus providing a novel tool to elucidate not only anatomy but also ecology and biology of ancient tetrapods.

## Introduction

Burrowing is a widespread adaptation in terrestrial mammals and is highly beneficial for brooding, predator avoidance and protection from extreme climates [1], [2]. Abundant fossilised burrow casts immediately after the Permo-Triassic boundary in southern Africa suggest that fossorialism was widely developed in many tetrapods more than 250 million years ago [3]–[9]. As this period is characterised by harsh climatic conditions, the disproportionate density of these ichnofossils in the Karoo reflects the survival strategy adopted by many tetrapods [10], [11]. A few of these lithified burrow infillings contain fossils that provide crucial information on ancient vertebrate ecology [4]–[7], [12]. While dens provided a stable and hospitable environment throughout the year [2], evidence suggests a period of dormancy in many of their inhabitants, probably elicited by resource scarcity [9], [12]–[14]. Despite the relative abundance of burrow casts, documented burrow-burrower associations remain rare, obfuscating the fossorial lifestyle of these animals.

The variety of burrow cast morphologies in Karoo rocks suggests that these retreats were excavated for different purposes. Several fossil therapsids discovered in curled-up positions [4], [9], [13], [15], [16] were interpreted as animals resting in confined spaces such as burrows. This suggestion was eventually corroborated by the discovery of the Early Triassic cynodont *Thrinaxodon* entombed in a burrow in a similar posture [4] and interpreted as an indication of seasonal dormancy [17]. An aestivating behaviour in therapsids has also been suggested from analyses of bone microstructure for a few taxa [13].

**Figure 1:**
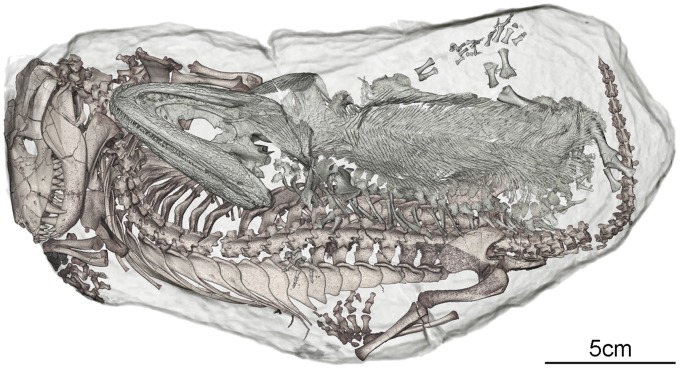
Upper-side 3D rendering of the content inside the burrow cast BP/1/5558 in semi-transparency. *Thrinaxodon liorhinus* (in brown; BP/1/7199) is lying on its ventral side; *Broomistega putterilli* (in grey; BP/1/7200) deposited upside down on the right side of the *Thrinaxodon*.

**Figure 2:**
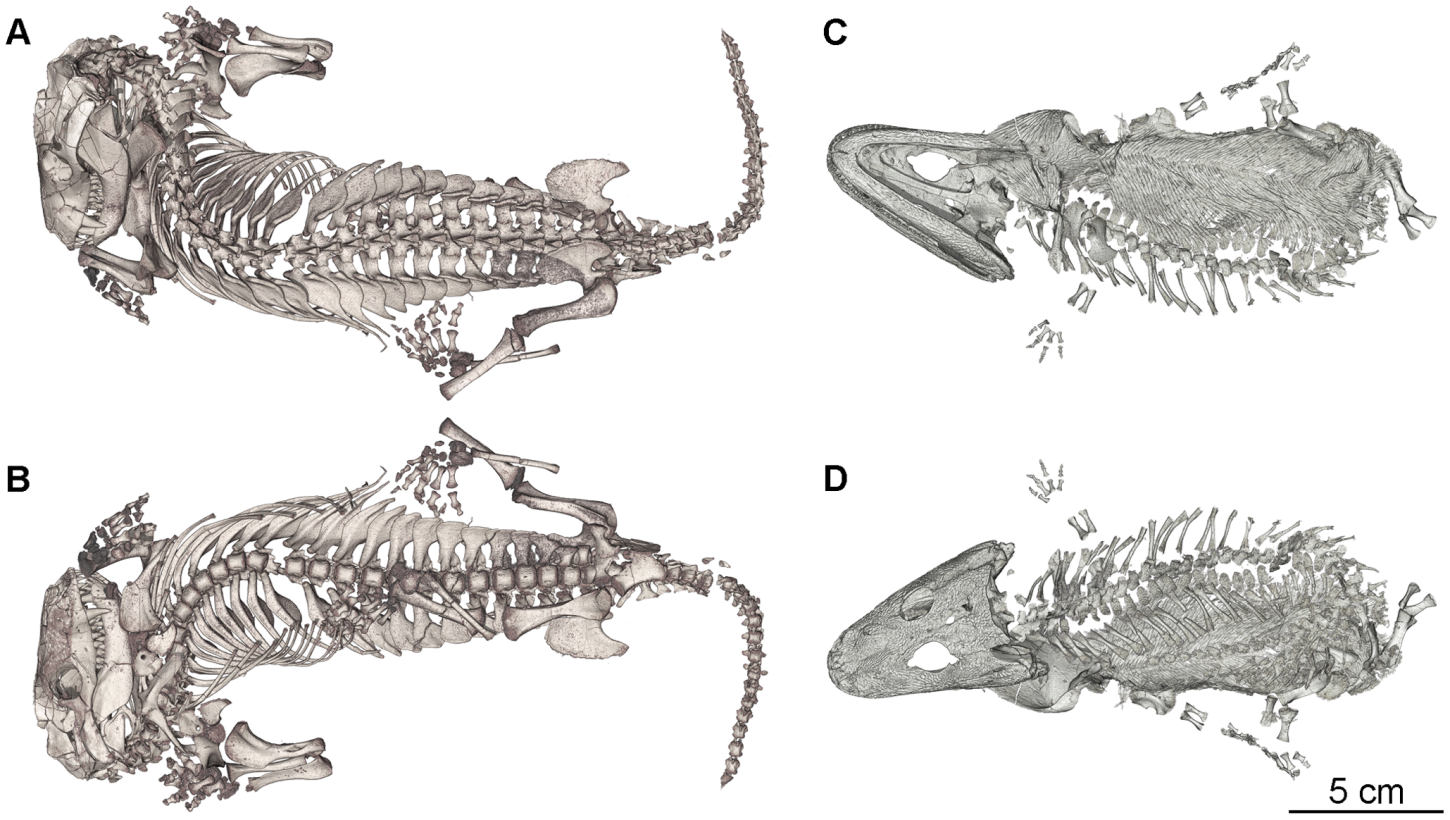
3D rendering of the two specimens from the burrow cast BP/1/5558. **A, B.** *Thrinaxodon liorhinus* (BP/1/7199) in dorsal (A) and ventral (B) views. **C, D.** *Broomistega putterilli* (BP/1/7200) in ventral (C) and dorsal (D) views.

**Figure 3:**
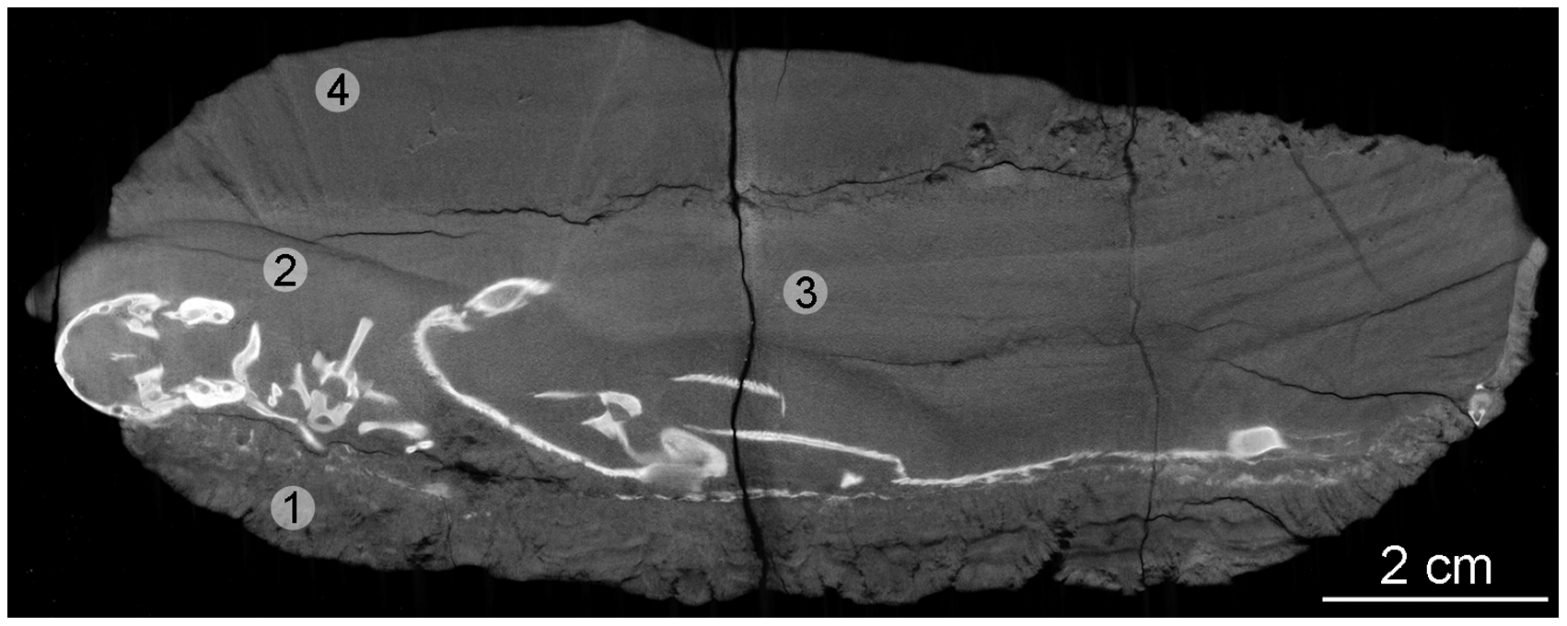
Virtual longitudinal vertical section of the burrow infill. The section clearly shows the 4 phases of sedimentary infill (1–4) with the specimens lying on the basal bed. (1) Bottom disrupted layer. (2) First pulse of the flooding event layer of massive sandstone. (3) Second pulse of the flooding event layer of sub-parallel bedding structures. (4) Overlying unit of fine sandstone.

Recent developments in synchrotron imaging have enabled investigation of burrow casts in a non-destructive way (see Methods). Using facilities at the European Synchrotron Radiation Facility (Beamline ID17, ESRF, Grenoble, France), we scanned an unprepared fossilised burrow cast (BP/1/5558, stored at the Evolutionary Studies Institute, Johannesburg) unearthed from Early Triassic rocks of the Karoo Basin. Remarkably, the synchrotron data reveal a unique association of two complete skeletons lying adjacent to each other: a therapsid cynodont *Thrinaxodon liorhinus*, and a temnospondyl amphibian *Broomistega putterilli* (Figures 1 and 2 and Movie S1), both embedded in the same sandstone bed (Figure 3). This new material creates the potential for investigating behavioural interaction of two unrelated taxa. Reasons for their association are discussed based on direct and circumstantial evidence, leading to the conclusion of a commensal relationship beneficial for the temnospondyl.

## Materials and Methods

The burrow cast (BP/1/5558) resides in the collection of the Evolutionary Studies Institute (University of the Witwatersrand, Johannesburg, South Africa). It was collected from the lower *Lystrosaurus* Assemblage Zone (Harrismith Member of the Normandien Formation) on Admiralty Estates at the base of Oliviershoek Pass (KwaZulu Natal Province, South Africa). At its initial discovery in 1975 by J. Kitching, the specimen showed only a small portion of a fossilised skull tentatively attributed to the genus *Thrinaxodon*. It was broken into two parts to investigate its contents, which led to the conclusion that the burrow cast encased postcranial material. The cast consists of a weathered oblong terminal chamber. As its external surface was eroded to some extent, it is not possible to comment on the presence or absence of scratch marks. The remaining, incomplete terminal chamber is 281 mm long and has a diameter ranging from 96 to 150 mm. As such, it is slightly smaller than a previously described *Thrinaxodon* burrow [4]. The burrow cast (BP/1/5558) comprises silty fine-grained sandstone with a micritic cement, possibly as a result of paedogenesis or early diagenesis.

The specimen was scanned on the ID17 beamline of the ESRF. The scan was performed using a monochromatic beam of 96 keV and a taper fiber optic, producing data with isotropic voxels of 45.5 microns. We used the attenuation protocol [18] and a sample/camera distance of 5 m in order to obtain some propagation phase contrast effect. The axis of rotation was put on the right side of the field of view in order to double the lateral reconstructed field of view using the so-called half-acquisition protocol [18]. Each sub-scan was reconstructed from 4000 projections of 0.5 s each and covered 7 mm vertically. Displacement of 3.5 mm between each sub-scan was used in order that each slice was acquired two times and averaged to increase final reconstruction quality.

In order to improve data quality and contrast, a single distance phase retrieval process [19] coupled with a 3D unsharp mask of the reconstructed volume were used. This approach improves significantly the signal to noise ratio and general contrast, without losing resolution, making 3D segmentation and rendering much more efficient than with absorption data or propagation phase contrast in edge detection mode only.

After the complete reconstruction process, volume processing and rendering was undertaken using the software VGstudiomax 2.1 (Volume Graphics, Heidelberg, Germany). The segmentation was performed at the VIP lab of the Palaeosciences Centre at the University of the Witwatersrand, using semi-automatic 3D region growing tools. When this tool did not permit complete extraction (e.g. too low contrast between the bone and the matrix or too high fracture level), missing parts were added slice by slice using manual segmentation as necessary.

No permits were required for the described study, apart from a temporary export permit from the South African Heritage Resources Agency (SAHRA) to transport the specimen to the ESRF, and the study complied with all relevant regulations.

## Results

The two entombed specimens represent two different genera already known from the fossil record. BP/1/7199 bears unambiguous features associated to the cynodont *Thrinaxodon liorhinus*: complex posterior postcanines with a main cusp and anterior and posterior accessory cusps in line and cingular cuspules; a relatively large dentary with a well-developed coronoid process [20]. The temnospondyl (BP/1/7200) displays diagnostic characters that allow its identification within Rhinesuchidea: deep otic notch with subparallel lateral margins; craniofacial ornamentation consisting mostly of pits with thin walls lacking prominent knots at the wall junctions; frontals excluded from orbital borders; muscular crest of the parasphenoid situated behind the posterior ends of the pterygo-parasphenoid sutures; the vomer forms most of the medial border of the choana and extends backwards to the level of the palatine tusk; supratemporal included in the margin of the otic notch [21]. Within the Rhinesuchidae, BP/1/7200 manifests characters of *Broomistega putterilli*: muscular crests of the parasphenoid located far behind the pterygo-parasphenoid suture; shallow oblique crest of the pterygoid, extending to only half the depth of the occiput; long and shallow subotic process of the exoccipital. The juvenile status of the specimen is indicated by the absence of shagreen on the palate [21].

Within the burrow, the *Thrinaxodon* specimen is positioned on the floor with the *Broomistega* alongside, but overlying the right side of the *Thrinaxodon*. It is clear that the burrow was filled by four discrete sedimentary events (Figure 3). The basal infill, on which the specimens are lying, is a relatively thin layer and the disorganised nature of the bedding suggests that it was disrupted, likely by trampling activity of the burrow occupants (Figure 3, region labelled *1*). The second unit is comparatively more massive and covers most of the animal remains (Figure 3, region labelled *2*). Its irregular surface draping the animals testifies to a high energy and rapid deposition. The third filling event covers the rest of the animals and shows sub-parallel bedding structures (Figure 3, region labelled *3*). The fourth, an overlying unit with a basal pebble lag, was deposited subsequent to the inundation that buried the specimens and its fining upward trend with a basal pebble lag reflects waning energy in a final pulse of sediment deposition (Figure 3, region labelled *4*).

Both specimens are almost fully articulated and preserve their individuality, with only a few minor postmortem bone displacements (Figure 2). The whole specimen (skeleton and burrow cast) is crossed by a crack, notably going through the pectoral girdle of the *Broomistega* (e.g., clavicles on Figure 1) and the vertebral column and hindlimbs of the *Thrinaxodon* (e.g., proximal part of the tibia-fibula from the right hindlimb; Figure 1). It was caused by cracking open the rock when it was discovered in order to confirm the presence of fossilised bone inside.

Within the burrow cast, the *Thrinaxodon* is lying dorsal surface up and shows an unnatural neck angulation (Figure 2 A and B). The distal ends of several ribs on the left side are broken off, but all of the broken tips remain aligned to the long axis of their respective rib bodies. Rupture of fresh bone results in spiral fractures with ragged edges, while smooth plane surfaces are indicative of postmortem compression, or are produced during the excavation/or preparation process [22]–[25]. Since these cracked ribs are characterised by smooth planes perpendicular to the long axis of the bone, we suggest they likely resulted from postmortem compression as the specimen was not excavated or physically prepared. The only missing elements of the skeleton are the posteriormost caudal vertebrae and most of the phalanges of the left manus. As the broken tip of the tail and the left manus are exposed on the surface of the burrow cast, their absence is likely attributable to weathering of the structure. Both hindlimbs are extended, the left below the body, the right to the side. Both fibulae and a couple of vertebrae are slightly displaced from anatomical position. The nature of the flexion exhibited by both forelimbs at the side of the skull conveys a natural resting position.

**Figure 4:**
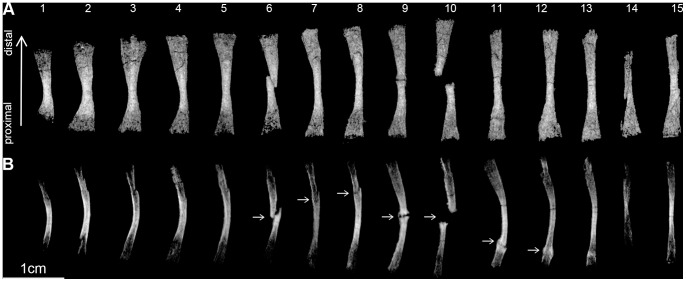
Right rib series of the *Broomistega putterilli*. **A**, 3D rendering of the rib series in dorsal view with individual ribs numbered from anterior to posterior (1 to 15). **B,** virtual radiography of the rib series in anterior view. The series of ribs display ante-mortem fractures (white arrows in B), some of which exhibit healing (ribs 9 to 12), including pseudoarthroses.

The *Broomistega* was buried supine, lying ventral surface up (Figure 1). Its skeleton is tilted, as it was partly propped up by the right flank of the *Thrinaxodon*. As a result, its postcranium, except the vertebrae, have settled a short distance away from the *Thrinaxodon* during fossilisation (Figure 2C, D). The skeleton is complete save for a few phalanges from the right pes. As this pes is exposed on the surface of the burrow cast, the missing bones were likely weathered away. This specimen, which represents the first reasonably complete rhinesuchid skeleton, notably shows that the manus has four digits and the skin has a complicated scale pattern. There are seven consecutive broken ribs on the right side of the *Broomistega* specimen (Figure 2 and 4). The fact that several of the fractures exhibit ragged edges (e.g., rib #6, Figure 4) indicates breakage while the bone was fresh [22]–[25]. The concomitant presence of healing structures (i.e., a bony callus) in some of the ribs confirms that the fractures were antemortem. At first glance, different degrees of healing may indicate fractures resulting from multiple traumas. However, the fractures are aligned in a diagonal line, likely indicating a single crushing trauma. The more posteriorly situated caudal ribs 11 and 12 are fractured near their distal ends. These fractures are well healed with new bone (i.e. a fracture callus, white arrows on Figure 4B) bridging the gaps, perhaps because the dorsal muscles stabilised the broken ends. Ribs 8, 9, 10, 11 and 12 have abundant fracture callus. Pseudarthroses near the broken edges suggest failure of the fractures to heal properly. These injuries are near the midshaft, where the ribs are most flexible in dorsoventral compression and where they would converge and separate a great deal during locomotion. Rib 7 and the more cranial ribs show slight expansion and increased density consistent with an incomplete (or greenstick) fracture where the ribcage is protected by the upper limb musculature. Ribs 13 to 15 present some structures that might be interpreted as the result of fractures, but unfortunately the scan resolution prevents definitive diagnoses. All of these injuries are consistent with a single crushing injury to the right thorax that the animal survived for at least several weeks, as evidenced by the degree of healing.

Two symmetrical circular holes are present anterior and posterior to the left orbit of *Broomistega* (Figure 2D). These holes have diameters of 3 mm and are spaced 16.5 mm apart. There are no other discernible marks on the remains of *Broomistega*.

## Discussion

*Broomistega* is the only rhinesuchid amphibian to have survived into the Triassic when it remained an uncommon component of the fauna [21]. Large spaces between the mineralised diaphyses of its limb bones, coupled with the absence of ossified carpal or tarsal bones and the poor state of vertebral ossification, as well as its pristine preservation, indicate that the skeleton of this specimen had a substantial cartilaginous component. These features suggest that this *Broomistega* specimen lived a primary aquatic lifestyle [26]. Besides this new specimen, *Broomistega* is known from three craniodental specimens, only one of which (BP/1/3241) is associated with a few postcranial remains [21]. While both sets of postcranial material (BP/1/3241 and BP/1/7200) exhibit poor ossification, crania associated with these specimens are approximately half the size of the holotype cranium (TM 184, stored at the Ditsong National Museum of Natural History, Pretoria). Temnospondyls increase in size during life [27], and larger specimens are always more ossified [28]. Therefore, we interpret the incomplete postcranial ossification as indicative of young ontogenetic age of the new specimen (BP/1/7200). Synchrotron data reveal seven consecutive partially healed broken ribs in *Broomistega* (Figure 4), resulting from a single crushing event that occurred several weeks before death. The healing process confirms that this trauma was not lethal, but certainly the animal’s locomotion and respiration would have been adversely affected.

Sediment encasing the skeletons suggests a high-energy burrow filling episode. The skeletons are exquisitely preserved in anatomical articulation and natural body position (Figure 2), and are not comingled (Figure 1), which indicates that the animals were buried with intact soft tissue, including the skin, and were possibly even alive [8]. Based on its morphology and anatomical characters, it is evident that *Broomistega* was unable to burrow. Evaluation of *Thrinaxodon* anatomy suggesting it was an active burrower remains an open question. However, this specimen, together with the other skeletons discovered in a burrow [4], demonstrates that it occupied burrows. As the juvenile *Broomestiga* was primary aquatic, *Thrinaxodon* was likely the primary occupant and most likely the excavator of the burrow.

Interspecific shelter sharing associations among extant vertebrates are uncommon in confined spaces, such as a den or underground burrow [2]. Several possibilities may explain this unusual aggregation of a terrestrial therapsid with a semi-aquatic rhinesuchid. We excluded the accidental hypothesis: the small diameter of the *Thrinaxodon* burrow [4] effectively excludes the possibility that the relatively large *Broomistega* was randomly washed in by a flood event. A prey-predator relationship is also unlikely since both skeletons are almost fully articulated and undisturbed, meaning it is implausible that one may have fed on the other. We considered the two holes present above the left orbit of the *Broomistega* as punctures (i.e., resulting of a bite). These holes are 16.5 mm apart while the intercanine distances in the *Thrinaxodon* specimen are 13 mm and 8 mm for the upper and lower canines, respectively. Despite a slight deformation of the skull of the *Thrinaxodon* specimen, the absence of clear matching of the canines with these holes, essentially excludes the possibility that these are canine bite marks made by the cynodont. The absence of other marks on the skull and mandibles of the *Broomistega* makes it difficult to interpret these holes as bite marks by another predator. On the better-preserved right side of the skull of the *Broomistega* specimen, there is a trough running along the margin of the skull table, reaching the posterior margin of the orbit and continuing for a short distance anterior to the orbit. This rare feature is distinct from the sensory canals system, and was occasionally described in other juvenile temnospondyls such as the Australian *Lapollopsis nana* [29]. The holes on the left side of the skull are located in this temporal trough, which corresponds to a local thinning of the bones (i.e. supratemporal, squamosal and postorbital posteriorly and prefrontal anteriorly). While the origin of these holes is not ascertained, the fact that they are located in a weakened zone of the skull means they could have resulted from diagenetic dissolution or could be pathological. Unfortunately, the resolution of the scan does not allow further investigation.

We finally considered that the *Broomistega* could have been dragged into the burrow by the *Thrinaxodon*, reflecting food-hoarding behaviour. However, perishable food hoarding in burrows by extant animals is rare, especially in hot environments favouring rapid decomposition [30]. The absence of a clear association of the puncture-like marks on the skull of *Broomistega* with the canines of the *Thrinaxodon,* combined with perishable food hoarding being an uncommon behaviour disfavour the prey-predator hypothesis.

Multi-taxa accumulations of vertebrates are usually elicited by external factors such as foraging, predator avoidance, or harsh climatic conditions [2], [31]. In modern terrestrial environments, animals are known to occupy burrows made by other taxa if: a) the burrow is abandoned [32]; b) they can chase away the host [32]; or c) the host tolerates their presence [32]–[34]. Extant amphibians, especially juveniles, are known to temporarily use terrestrial burrows as a protective retreat [31], [35], which we conclude to be the most probable strategy adopted by the injured juvenile *Broomistega*, given the unlikely scenarios excluded above.

The fact that the *Broomistega* was not savaged by the presumed *Thrinaxodon* host suggests that the latter either tolerated the presence of the amphibian intruder or was not able to evict it (i.e., either through deep torpor or because of death). As the *Thrinaxodon* skeleton is articulated, its death prior to the flooding event would imply a certain degree of mummification and stiffness of the carcass. The pleurothotonus posture (sideways spinal curvature) is, however, too heavily marked to have been caused by physiological processes (e.g., death throes) or abiotic desiccation [36]. It more likely resulted from compression of a flaccid body against the terminal wall of the burrow. This is corroborated by the posture of the *Broomistega*, which was pushed onto the back of the *Thrinaxodon* by the current flow [36], and may also account for the curvature of both tails toward the ends of the burrow (Figure 1 and 2D). Consistent with the preservation of soft tissues and complete articulation of the skeleton, the *Thrinaxodon* either died only shortly before the flooding, but after rigidness associated with *rigor mortis* lapsed, or its death was directly related to the flooding. As the timing of the “*Thrinaxodon* died first” hypothesis would imply exceptional circumstances, we favour tolerance of the *Broomistega* by the cynodont.

The timing of the death of the *Broomistega* was also constrained as we consider that it may have crawled in the burrow voluntarily and that its complete state of preservation implied presence of soft tissues. Its curved spinal posture is not compatible with post-mortem stiffness, suggesting that its body also was flaccid at the time of the flooding. Thus, the *Broomistega* specimen also appears to have died either slightly before the flooding event or during the actual inundation. In both cases, its posture, lying ventral-up on the flank of the *Thrinaxodon* specimen was likely the result of current flow rather than a perimortem physiological process [36].

Cases of tolerance by a host exist amongst extant tetrapods, especially during harsh climatic conditions [31], [32]. When the intruder provides an antipredator advantage to the host (i.e., enhances vigilance), the latter willingly shares the den, thereby increasing its probability of survival [33]. However, these cases involve clusters of burrows (i.e., each with their own opening) rather than connecting burrows (i.e., shared openings), and different species occupy different burrows within the cluster [33]. Cases of non-beneficial co-habitation have been reported, but are extremely rare: for instance, whilst hibernating, European badgers of the Białoweża Primeval Forest (eastern Poland) tolerate the presence of raccoon dogs in their setts [34]. As the Early Triassic climate of southern Africa was arid, torpor during the warmest and driest part of the year (i.e., aestivation) has been envisioned as an explanation for the presence of several therapsid skeletons entombed in burrows, especially those displaying a resting or curled-up position [9], [12]–[14], [16]. Studies of aestivation in extant mammals indicate that prolonged inactivity and fasting reduces digestive organ mass [37], and causes lines of arrested growth (LAGs) in limb bones [38]. While the impact of such inactivity on organs is virtually impossible to retrieve in fossils, LAGs have been observed in the long bones of several therapsids, some of which have been discovered in curled-up positions [13]. Studies of *Thrinaxodon* bone histology have not revealed LAGs, concluding that a continuous growth rate driven by a lifestyle avoiding high climatic fluctuation was more likely [13]. A fossorial lifestyle can provide an environment with stable temperature and humidity throughout the year. Torpor, self-induced in mammals by extreme conditions, does not always consist of an uninterrupted period of dormancy [37]. Indeed, several mammals, such as the woodchuck *Marmota monax* [39], experience short periods of dormancy [37], which may last only a few days. As a result, LAGs are absent in the limb bones of the woodchuck and the only potential histomorphological structure is a series of indistinct growth arrest lines on the periphery of bone cross sections [13]. LAGs can thus be completely absent in taxa having limited hibernation or short but repetitive aestivation periods [40].

Deprivation of food and water in arid environments often leads to some states of torpor in many extant mammals that do not considerably decrease their metabolic rate [37]. The use of a secluded place, such as a burrow, helps the animal to reduce its body temperature to contend with the lack of resources [37]. The unusual instance of cohabitation manifested in specimen BP/1/5558, in addition to the numerous specimens of *Thrinaxodon* discovered in curled-up positions [4], [9], [13], [15], suggests that this animal had retreated into its burrow for a period of dormancy. However, the absence of histomorphological markers indicative of arrested appositional bone growth, suggests that these periods were relatively short in duration. As torpor is viewed as a plesiomorphic character in mammals [41], it is more likely that metabolic plasticity existed in this mammal forerunner rather than the specialised metabolism of a hibernator. The capacity to escape hazardous climatic conditions in a burrow and to survive deprivation of vital resources certainly contributed to the success of small to medium-sized cynodonts across the PT crisis [10], [42].

Interspecific cohabitations are difficult to interpret in the extant record; while benefits for the intruder seem straightforward, it is not always clear why the host tolerates intruders. This difficulty is exacerbated by a fossil record that provides only single snapshots of time. The abundance of ichnofossils in the Karoo enables the testing of hypotheses related to inter- and intraspecific behavior. Recent developments in synchrotron imaging offer new opportunities to visualise burrow contents that facilitate a more thorough understanding of the lifestyle of ancient tetrapods and the reasons for their survival.

Technological development made in several synchrotrons around the world over the past decade, allow palaeontologists to investigate larger fossilised remains with great accuracy. This experiment demonstrated how large specimens can be studied in a non-destructive way within their sedimentological context. This is of tremendous importance for the study of fossilised burrows.

## Supporting Information

Movie S1 **Animation showing the result of the 3D segmentation of both specimens within the burrow in upper lateral view.** Within the burrow (BP/1/5558), the *Thrinaxodon* specimen (in brown; BP/1/7199) is positioned on the burrow floor with the *Broomistega* (in grey; BP/1/7200) alongside, but overlying the right side of the *Thrinaxodon*.
(MOV)
